# Microplastic ingestion ubiquitous in marine turtles

**DOI:** 10.1111/gcb.14519

**Published:** 2018-12-04

**Authors:** Emily M. Duncan, Annette C. Broderick, Wayne J. Fuller, Tamara S. Galloway, Matthew H. Godfrey, Mark Hamann, Colin J. Limpus, Penelope K. Lindeque, Andrew G. Mayes, Lucy C. M. Omeyer, David Santillo, Robin T. E. Snape, Brendan J. Godley

**Affiliations:** ^1^ Marine Turtle Research Group, Centre for Ecology and Conservation University of Exeter Penryn UK; ^2^ College of Life and Environmental Sciences: Biosciences University of Exeter Exeter UK; ^3^ Marine Ecology and Biodiversity Plymouth Marine Laboratory Plymouth UK; ^4^ Faculty of Veterinary Medicine Near East University Nicosia North Cyprus Turkey; ^5^ Society for Protection of Turtles Kyrenia North Cyprus Turkey; ^6^ North Carolina Wildlife Resources Commission Beaufort North Carolina; ^7^ College of Science and Engineering James Cook University Townsville QLD Australia; ^8^ Department of Environment and Science Threatened Species Unit Brisbane QLD Australia; ^9^ School of Chemistry University of East Anglia, Norwich Research Park Norwich UK; ^10^ Greenpeace Research Laboratories, School of Biosciences, Innovation Centre Phase 2 University of Exeter Exeter UK

**Keywords:** anthropogenic debris, marine debris, marine plastic, marine turtle, microplastics, plastic pollution

## Abstract

Despite concerns regarding the environmental impacts of microplastics, knowledge of the incidence and levels of synthetic particles in large marine vertebrates is lacking. Here, we utilize an optimized enzymatic digestion methodology, previously developed for zooplankton, to explore whether synthetic particles could be isolated from marine turtle ingesta. We report the presence of synthetic particles in every turtle subjected to investigation (*n* = 102) which included individuals from all seven species of marine turtle, sampled from three ocean basins (Atlantic [ATL]: *n* = 30, four species; Mediterranean (MED): *n* = 56, two species; Pacific (PAC): *n* = 16, five species). Most particles (*n* = 811) were fibres (ATL: 77.1% MED: 85.3% PAC: 64.8%) with blue and black being the dominant colours. In lesser quantities were fragments (ATL: 22.9%: MED: 14.7% PAC: 20.2%) and microbeads (4.8%; PAC only; to our knowledge the first isolation of microbeads from marine megavertebrates). Fourier transform infrared spectroscopy (FT‐IR) of a subsample of particles (*n* = 169) showed a range of synthetic materials such as elastomers (MED: 61.2%; PAC: 3.4%), thermoplastics (ATL: 36.8%: MED: 20.7% PAC: 27.7%) and synthetic regenerated cellulosic fibres (SRCF; ATL: 63.2%: MED: 5.8% PAC: 68.9%). Synthetic particles being isolated from species occupying different trophic levels suggest the possibility of multiple ingestion pathways. These include exposure from polluted seawater and sediments and/or additional trophic transfer from contaminated prey/forage items. We assess the likelihood that microplastic ingestion presents a significant conservation problem at current levels compared to other anthropogenic threats.

## Graphical abstract

Knowledge of the incidence and levels of synthetic particles in large marine vertebrates is lacking. Here, we explore whether synthetic particles could be isolated from marine turtle ingesta and report the presence in every turtle subjected to investigation including individuals from all the seven species of marine turtle, sampled from three ocean basins. Most particles were fibres in lesser quantities were fragments/microbeads and were a range of synthetic materials. Synthetic particles isolated from species occupying different trophic levels suggest the possibility of multiple ingestion pathways. We assess the likelihood this presents a significant conservation problem at current levels.

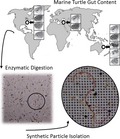

## 1 INTRODUCTION

Plastic debris is ubiquitous in the marine environment (Rochman et al., 2015). It is estimated that 4.8–12.7 million tonnes of plastic waste could be entering the marine environment annually, contributing to an estimated five trillion pieces of plastic in the surface waters of the global seas (Eriksen et al., 2014; Jambeck et al., 2015). Recently, there has been a growing concern regarding “microplastics,” which are defined as plastic particles <5 mm. Due to their high abundance and bioavailability, microplastics have been considered as a pollutant in their own right (Andrady, 2011; Cole, 2014).

Primary microplastics are most commonly associated with exfoliators in cosmetic products, or preproduction nurdles but can also result from “microbead” use in biomedical applications, air‐blasting technology, automotive tyre wear or fibres from the breakdown of clothing (Cole, Lindeque, Halsband, & Galloway, 2011; Derraik, 2002; Napper & Thompson, 2016; Napper, Bakir, Rowland, & Thompson, 2015; Nelms et al., 2017). Secondary microplastics are derived from the disintegration of larger plastic items (“macroplastics”) within marine systems through wave action, UV radiation exposure and physical abrasion as the items are moved by wave action or washed over shorelines. The cumulative effects of these physical, biological and chemical processes reduce the structural integrity of the plastic and result in fragmentation of the items into smaller, eventually microscopic particles (Browne, Galloway, & Thompson, 2007).

Ingestion of microplastics is now being reported in a number of marine invertebrate species (Cole et al., 2014; Dawson et al., 2018; Foley, Feiner, Malinich, & Höök, 2018; Long et al., 2017; Setälä, Fleming‐Lehtinen, & Lehtiniemi, 2014; Watts et al., 2014; Wright, Rowe, Thompson, & Galloway, 2013). The possible physiological and ecological effects of ingestion for these species is beginning to be understood; for example microfibre ingestion in crabs can affect food consumption and energy balance and ingestion of microscopic unplasticized polyvinylchloride reduces growth and energy reserves in marine worms (Watts, Urbina, Corr, Lewis, & Galloway, 2015; Wright et al., 2013). Descriptive reports are also starting to appear for vertebrates such as fish (Collard, Gilbert, Eppe, Parmentier, & Das, 2015; Foley et al., 2018; Güven, Jovanovi, & Erkan Kıdey, 2017; Lusher, McHugh, & Thompson, 2013; Rochman et al., 2015; Stolte, Forster, Gerdts, & Schubert, 2015) and marine mammals (Fossi et al., 2016, 2012; Lusher, Hernandez‐milian, Berrow, Rogan, & Connor, 2018; Nelms, Galloway, Godley, Jarvis, & Lindeque, 2018).

Knowledge relating to the incidence of microplastic (<5 mm) ingestion in marine turtles still remains very limited, despite records of all seven species of marine turtles ingesting macroplastics (>5 mm) (Boyle & Limpus, 2008; Hoarau, Ainley, Jean, & Ciccione, 2014; Lynch, 2018; Nelms et al., 2016; Schuyler, Hardesty, Wilcox, & Townsend, 2014; Yaghmour et al., 2018) and the creation of global risk maps aiding in the identification of interaction hotspots (Schuyler et al., 2015). The only exception is the isolation of seven microplastic particles (<5 mm) from the gut contents of two green (*Chelonia mydas*) turtles from the Great Barrier Reef (Caron et al., 2018) and recent accounts relating to stranded posthatchlings from the Atlantic (White et al., 2018).

Rising concerns regarding global impacts of microplastic pollution on marine wildlife mandates a reliable and comparable detection protocol (Nelms et al., 2016). Here, alongside investigation of macroplastic ingestion (>5 mm), we develop a methodology to explore whether synthetic particles (<5 mm) could be isolated from marine turtle ingesta. We sought to: (a) identify the extent of microplastic ingestion in all species of marine turtles; and (b) explore the polymer type of any ingested particles.

## 2 MATERIALS AND METHODS

### 2.1 Study sites

**Figure 1:**
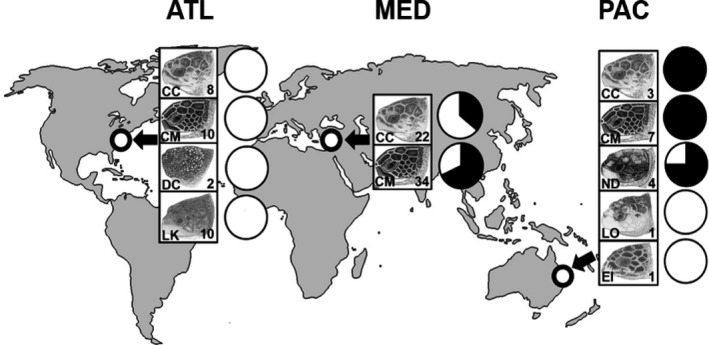
Study sites and number of each species sampled; Embedded pie charts of proportion of individuals with macroplastic ingestion (%); white = absent, black = present. Left to right: Atlantic (North Carolina, USA), Mediterranean (Northern Cyprus), Pacific (Queensland, Australia). Species codes: CC = loggerhead turtle (*Caretta caretta*), CM = green turtle (*Chelonia mydas*), DC = leatherback turtle (*Dermochelys coriacea*), LK = Kemp's ridley turtle (*Lepidochelys kempii*). ND = flatback turtle (*Natator depressus)*, EI = hawksbill turtle (*Ertmochelys imbricata*) and LO = olive ridley turtle (*Lepidochelys olivacea).* Sea turtle skull figures used with permission of WIDECAST; original artwork by Tom McFarland

The study was conducted in three ocean basins using both stranded and bycaught animals (*n* = 102; all seven marine turtle species. In the Mediterranean basin (MED), samples were collected from Northern Cyprus where stranded and bycaught green (*C. mydas*) and loggerhead (*Caretta caretta*) turtles are common. In the Atlantic basin (ATL), samples were collected from North Carolina, USA which experiences strandings of green, loggerhead, Kemp's ridley (*Lepidochelys kempii*) and leatherback (*Dermochelys coriacea*) turtles. Finally, the Pacific basin (PAC) with samples provided from Queensland, Australia which included stranded and bycaught posthatchling green, loggerhead, flatback (*Natator depressus*), hawksbill (*Eretmochelys imbricata*) and olive ridley turtles (*Lepidochelys olivacea*) (Summarized in Supporting Information Table S1.; Figure 1).

### 2.2 Necropsy and gut content analysis

Animals were subject to necropsy and biometric parameters were taken (minimum curved carapace length (Bolten, 1999). The entire gastrointestinal tract was removed and initial contents were weighed and then rinsed through a 1 mm mesh sieve. The remaining matter in the sieve was emptied into trays for sorting with macroplastic removed and stored for later analysis. A 100 ml sample (approximately 5% of the total) of gut content residue and associated supernatant was collected from material that had passed through the 1 mm mesh sieve. This was later oven dried at 60°C for 24 hr to enhance the efficacy of homogenization in later steps of the process. Gut content residue samples were exposed to an optimized enzymatic digestion protocol that had been developed for use on zooplankton material by Cole et al., (2014). Digestion filters were then analysed under a digital stereo microscope (Leica M165C) and classified by type, colour and size. A subsample (*n* = 169) of these identified particles were analysed using FT‐IR spectroscopy (FT‐IR) (Supporting Information Figure S2). Extensive measures were taken to minimize possible sample contamination (For full details see Supporting Information Data S1 [Supplemental methods]).

## 3 RESULTS

### 3.1 Synthetic particle ingestion

**Figure 2:**
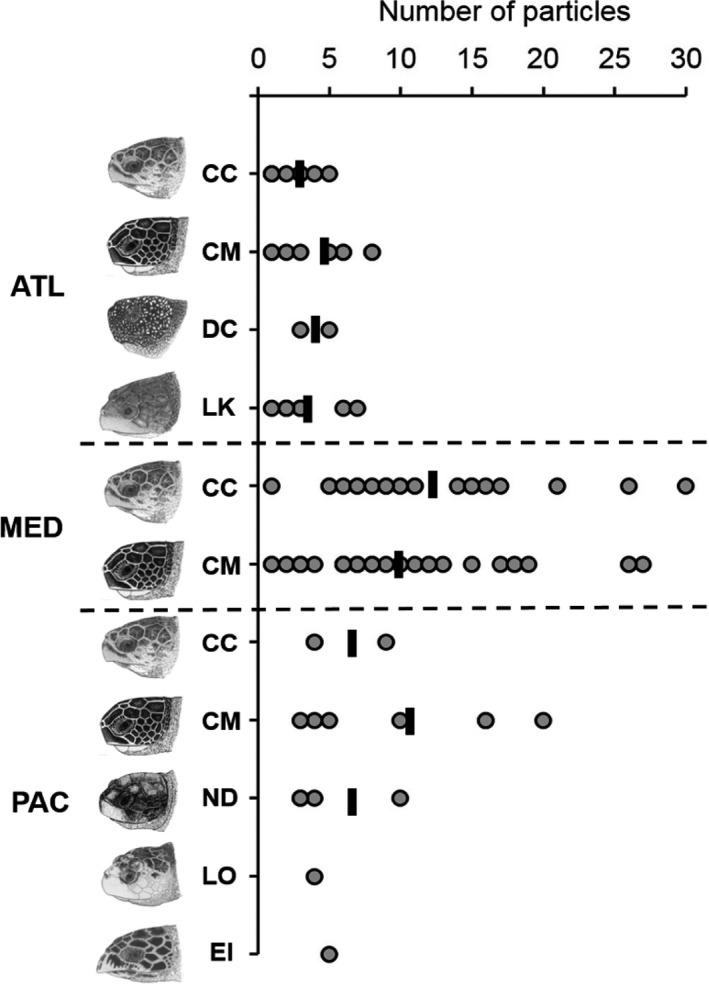
Synthetic microparticle ingestion in all species of marine turtles from three ocean basins. Total number of particles identified in each 100 ml subsample per species per ocean basin. Black line = mean number of particles. Note that 100 ml was analysed per animal irrespective of size, so the number of particles per animal should not be over‐interpreted. ATL = Atlantic (North Carolina, USA) loggerhead turtle (*Caretta caretta*,* n* = 8), green turtle (*Chelonia mydas*,* n* = 10), leatherback turtle (*Dermochelys coriacea*,* n* = 2), Kemp's ridley turtle (*Lepidochelys kempii*,* n* = 10). MED = Mediterranean (Northern Cyprus) loggerhead turtle (*n* = 22), green turtle (*n* = 34). PAC = Pacific (Queensland, Australia) loggerhead turtle (*n* = 3), green turtle (*n* = 7), flatback turtle (*Natator depressus*,* n* = 4), hawksbill turtle (*Eretmochelys imbricata*,* n* = 1) and olive ridley turtle (*Lepidochelys olivacea*,* n* = 1). Sea turtle skull figures used with permission of WIDECAST; original artwork by Tom McFarland

Synthetic particles (<1 mm) were identified in every individual (*n* = 102) of all seven species over the three ocean basins, with 811 particles isolated in total. This 100% incidence contrasts with highly variable occurrence rates of macroplastic (>5 mm) ingestion in some species in the study areas (range: 0%–100%) (Figure 1). Although sample sizes were small for some site‐specific species groups, there was a marked variability of incidence in synthetic particle ingestion among sites, with levels appearing higher in turtles from the Mediterranean (Figure 2).

### 3.2 Particle description

**Figure 3:**
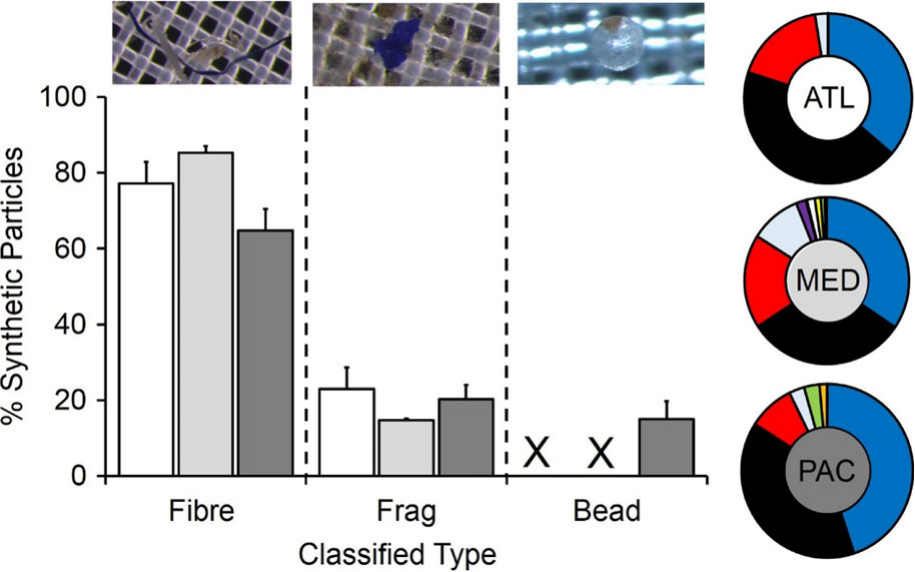
Type and colour of synthetic particles including microplastics identified from marine turtle gut content. Mean (±*SE*) percentage make‐up of each type (fibre, fragments, beads) isolated within the gut content residue samples from stranded turtles from the Atlantic (white), Mediterranean (light grey) and Pacific (dark grey). Colours categorized for fibrous synthetic particles ATL = Atlantic, MED = Mediterranean and PAC = Pacific. X = no detections

The type of particle varied among sites. The majority of these were classified as fibres at all three sites (ATL: 77.1%: MED: 85.3% PAC: 64.8%) and in lesser quantities were fragments (ATL: 22.9%: MED: 14.7% PAC: 20.2%) and microbeads (4.8%; PAC only) (Figure 3). Fibres spanned several of the 11 colour categories (ATL: 4/11; MED: 10/11; PAC: 6/11) but the large majority of fibres were blue or black in all sites (Blue: ATL: 36.3%; MED: 34.4%; PAC: 44.9%; Black: ATL: 43.7%; MED: 31.3%; PAC: 39.1) followed by red and clear fibres (Red: ATL: 17.5%; MED: 18.2%; PAC: 8.6%; Clear: ATL: 2.5%; MED: 9.9%; PAC: 2.9%) (Figure 3).

### 3.3 Polymer identification

A subsample of 20% (*n* = 169) of the isolated particles were tested using FT‐IR to determine their polymer composition (SupportingInformation Table S2). This analysis revealed the majority were synthetic materials (*n* = 160) (ATL: 100%; MED: 92.6%; PAC: 100%) with only a minority being naturally occurring materials such as natural rubber and plant protein (*n* = 9) (MED: 7.4%). In addition, not all synthetic materials comprised plastic polymers. Our spectral matches identified elastomers (MED: 61.2%; PAC: 3.4%) such as ethylene propylene diene monomer rubber (EPDM rubber), hydronated nitrile butadiene rubber (HNBR) and neoprene. We also identified woven synthetics (MED: 4.9%) such as polyaramid Kevlar^®^ and synthetic regenerated cellulosic fibres (SRCF), for example, rayon, viscose (ATL: 63.2%; MED: 5.8%; PAC: 68.9%). Of the confirmed true microplastics (ATL: 36.8%; MED: 20.7%; PAC: 27.7%), we identified the spectral characteristics of polyethylene (PE), ethylene propylene, polyester, with isolated microbeads being identified as polyacrylamide.

## 4 DISCUSSION

### 4.1 Synthetic particle ingestion in marine turtles

Here, we have shown that synthetic particles including microplastics (<5 mm) were present in every turtle, across all species and ocean basins sampled, even though not all individuals had ingested macroplastics. Sample sizes and methodology preclude in‐depth analysis here but ingestion may be generally higher in the MED basin than the wider ATL or PAC. Global models predict some of the world's highest concentrations of marine plastics in this basin (Cózar et al., 2014; Duncan et al., 2018; Eriksen et al., 2014; Suaria et al., 2016). Further, more exhaustive sampling is required to fully appraise interspecific and geographic differences.

Most particles isolated in our analysis were fibrous in nature. Indeed, fibres are now a prolific pollutant and are some of the most commonly observed in the natural environment; with numerous potential sources (Gago, Carretero, Filgueiras, & Viñas, 2018). In terms of colour, our results mirror studies on plankton ingestion, environmental seawater and sediments, with the majority of fibrous microplastics being predominately black, blue or red (Gago et al., 2018; Güven et al., 2017; Steer, Cole, Thompson, & Lindeque, 2017). Sources of synthetic fibres include microfibre shedding from the mechanical and chemical stresses undergone by synthetic fabrics (De Falco et al., 2018; Napper & Thompson, 2016), automotive tyre wear (Wagner et al., 2018) and degradation of cigarette filters and fragmentation of maritime equipment such as ropes and fishing nets (De Falco et al., 2018; Napper & Thompson, 2016). Synthetic fibre ingestion has been documented in filter feeding marine invertebrates such as mussels, clams and zooplankton and are thought to be in some cases mistaken for natural prey items (Davidson & Dudas, 2016; Mathalon & Hill, 2014). However, within marine turtles, due to the size of particles, ingestion is more likely to be through indirect mechanisms (ingestion pathways discussed further below) (Nelms et al., 2016).

Fragments were found as a minority in all three basins and microbeads were only identified in our samples from the Pacific Ocean. To our knowledge, this is the first isolation of microbeads from marine megavertebrates, being only identified in fish and planktonic gut content previously (Lusher, Welden, Sobral, & Cole, 2017; Peters, Thomas, Rieper, & Bratton, 2017; Setälä, Norkko, & Lehtiniemi, 2015; Steer et al., 2017; Tanaka & Takada, 2016). This could potentially be due to the foraging ecology of turtles sampled from the Pacific. Posthatchlings are known to be epipelagic surface dwelling unlike their neritic coastal counterparts (Bolten, 2003; Clukey, Lepczyk, Balazs, Work, & Lynch, 2017; Ryan et al., 2016) leading to a spatial overlap with surface floating microplastics.

### 4.2 Microplastic polymer identification

The polymer make‐up of marine plastic debris may aid in identifying possible sources, degradation, fate and reasons for ingestion (Jung et al., 2018; Nelms et al., 2018). The polymers identified through FT‐IR analysis reflect the recently reported polymer diversity globally described for microplastics (Gago et al., 2018). PE and polypropylene (PP) are some of the most abundant polymers found as pollutants worldwide (Gago et al., 2018; White et al., 2018). Furthermore, Suaria et al. (2016) identified 16 classes of synthetic material from the surface waters of the central‐western Mediterranean Sea. Within these classes, low‐density polymers such as PE and PP were again abundant, followed less frequently by polymers such as polyethylene terephthalate, polystyrene and polyamides which were also identified in the marine turtle gut content of this study. However, in our study, a large proportion of synthetic samples in the Mediterranean, belonged to the class of elastomers (e.g., EPDM Rubber, HNBR Rubber, Nitrile Butadiene Rubber). A major contributor to the presence of elastomers in the marine environment being tyre wear particles, with the majority of emission coming from road side run‐off (Wagner et al., 2018). Polyacrylamide microbeads described in our Pacific samples have been used in the past in drug delivery (El‐Samaligy & Rohdewald, 1982) and more recently for a number of biomedical applications such as encapsulation (Labriola, Mathiowitz, & Darling, 2017). Alternatively, these could originate from exfoliating agents in cosmetic products (Napper et al., 2015).

There are numerous challenges in studying microplastics in the environment including the analytical chemistry to identify particles (Comnea‐Stancu, Wieland, Ramer, Schwaighofer, & Lendl, 2017; Silva et al., 2018). Visual examination is the most common method used to identify microplastics. Although efficient, in situ and low cost, there are several limitations, such as the inherent difficulty in distinguishing microplastics from other small particles, e.g. natural or synthetic materials. Many potential microplastic fibres from the FT‐IR subsample in this study were identified with high spectral matches as cellulose‐based particles, despite their appearance under visual examination as microplastics. Indeed, this has begun to be reported elsewhere within the literature (Cai et al., 2017; Courtene‐Jones, Quinn, Gary, Mogg, & Narayanaswamy, 2017; Remy et al., 2015). For example, blue cotton indigo fibres from samples of waste water treatments plants can show close visual similarity to polyacrylic fibres (Dyachenko, Mitchell, & Arsem, 2017; Silva et al., 2018).

However, from further inspection of other digital photographs, individual spectra and high match qualities (over 80%–90%), we propose that these are SRCF such as viscose or rayon. Although originally derived from natural sources they undergo several chemical processes in regeneration to become reconstructed (Comnea‐Stancu et al., 2017; Gago et al., 2018). There are distinct differences between native and regenerated cellulose regarding their crystalline structure. These differences could affect their persistence in the marine environments, and hence their presence in marine turtle guts. Such SRCFs could represent a major fraction of fibres in the environment (Comnea‐Stancu et al., 2017). Future research should aim to build protocols to accurately interpret outputs, to be able to distinguish between SRCFs and other natural materials as it is clear that visual inspection alone is insufficient.

### 4.3 Ingestion pathways

There are multiple potential ingestion pathways. Firstly, the presence of synthetic particles in marine turtles could be due to environmental exposure to areas of contaminated sea water or sediments. Numerous studies have now identified microplastics in seawater worldwide creating potential exposure during foraging, nesting and migration (Critchell et al., 2015; Gago et al., 2018; van Sebille et al., 2015). Microplastics have also been shown to move from source to sediments (Gago et al., 2018), with low‐density plastics eventually reaching the seafloor though density‐modification, as a result of biofouling or integration into zooplankton faecal matter (Alomar, Estarellas, & Deudero, 2016; Andrady, 2011; Cole et al., 2016; Coppock, Cole, Lindeque, Queirós, & Galloway, 2017; Cózar et al., 2014; Van Cauwenberghe, Devriese, Galgani, Robbens, & Janssen, 2015). Many marine turtles are known to feed benthically, for example, benthic feeding loggerhead turtles actively rework sediments which are ingested along with their prey (Casale et al., 2008; Lazar et al., 2011; Preen, 1996).

Another pathway of exposure could be from particles in or on primary producers and sessile filter feeders, when the feeding ecology of hawksbill and green turtles is considered (Bell, 2013; Bjorndal, 1980; Obura, Harvey, Young, Eltayeb, & Brandis, 2010). For example, microplastics can adhere to the surface of seaweeds electrostatically binding to cellulose or retention facilitated by a mucus layer on the surface (Gutow, Eckerlebe, Giménez, & Saborowski, 2016) and sponges are known to ingest microplastics (Baird, 2016), creating a pathway of ingestion alongside dietary items.

Finally, synthetic particle presence in omnivorous life stages or species, especially loggerhead or ridley turtles, could originate through a pathway of trophic transfer from contaminated prey such as filter feeding invertebrates. Laboratory studies have shown trophic transfer of microplastics between invertebrates and within planktonic food webs (Dawson et al., 2018; Farrell & Nelson, 2013; Macali et al., 2018; Setälä et al., 2014). In addition, a recent study by Nelms et al. (2018) on grey seals (*Halichoerus grypus*) and wild‐caught Atlantic mackerel (*Scomber scombrus*) suggested that trophic transfer represents an indirect but potentially major pathway for any species whose feeding ecology involves the consumption of whole prey.

### 4.4 Potential impacts

We only tested a subsample of the gut content residue in our study and these represent a minimum count of the number of the gut burden. The total number of synthetic particles within the whole gut is likely to be the order of 20 times higher. This suggests that the total levels of ingestion per individual (whole gut) may be higher in marine turtles than large marine mammals. In a recent study focusing on cetaceans (*n* = 21), stranded and bycaught individuals were found to contain plastic particles ranging from one to 88 in whole digestive tract samples. These were composed of the majority fibres (83.6%) and the remaining were fragments (16.4%) (Lusher et al, 2018).

It remains unknown if and how these synthetic particles will impact turtles. Their size means they will pass through the gut lumen with relative ease (especially, for larger specimens) and therefore their presence does not lead to blockage or obstruction which is frequently reported in association with macroplastic ingestion (Ryan et al., 2016). Importantly, future work should focus on whether microplastics may be affecting aquatic organisms more subtly, for example, exposure to associated contaminants (heavy metals, persistent organic pollutants and polychlorinated biphenyls) and pathogens, or by acting at cellular or subcellular level (Critchell & Hoogenboom, 2018; Foley et al., 2018; Jovanović et al., 2018; Nelms et al., 2016; Velzeboer, Kwadijk, & Koelmans, 2014).

Due to successful application of the optimized enzymatic digestion protocol in marine turtles to confirm the presence and ingestion of suspected microplastics and other synthetic materials, we recommend this protocol for surveying other large marine vertebrate gut content or to be used in combination with other novel techniques newly proposed in the literature (Caron et al., 2018; Felsing et al., 2018; Herrera et al., 2018). The method has already been used to demonstrate the presence of microplastic ingestion in marine mammals (Nelms et al., 2018). When there is clear overlap between high levels of microplastic pollution and the presence of large marine vertebrates, the application of this technique could aid in the confirmation of this occurrence and whether overlap results in ingestion, and with careful work, at what magnitude. Similarly, the enzymatic digestion technique could be built into existing bioindicator protocols, which investigate macroplastic pollution, such as the Fulmar protocol (van Franeker & Law, 2015) and as such marine megavertebrates could serve as a bioindicators for both macro‐ and microplastics.

By adapting a methodology previously used on marine invertebrates, this study has revealed that marine turtles are interacting with this cryptic pollutant. Further research is required to help discern which microplastic ingestion pathways are significant and whether there are species and site‐specific variability in abundance and makeup of the particles ingested. Whilst these particles may be ubiquitous, and at higher levels than in marine mammals thus far surveyed, unless they play a role in amplifying exposure to associated contaminants, we suggest they are unlikely to present a significant conservation problem at current levels and are less of a concern than fisheries bycatch, the ingestion of macroplastics or entanglement in anthropogenic marine debris (Duncan et al., 2017; Nelms et al., 2016).
