# Supplementary material for: Microplastic ingestion ubiquitous in marine turtles
Source: Glob Chang Biol. 2018 Dec 4;25(2):744–52. doi: 10.1111/gcb.14519 (PMC6849705; doi:10.1111/gcb.14519)
Supplement: Supplementary file 1 [file GCB-25-744-s001.docx]

**Supporting Information**

| **Site** | **Species** | **n** | **CCL range** | **Date**  **Range** | **Macro-plastic ingestion (%)** | **Numbers of Particles** | | | | |
| --- | --- | --- | --- | --- | --- | --- | --- | --- | --- | --- |
|  |  |  | **(cm)** |  |  | **Elastomers** | **Woven** | **Plastics** | **SCRFs** | **Non-Syn.** |
|  | Green | 34 | 25-86 | 2011-16 | 68 | 22 | 2 | 12 | 3 | 3 |
| Northern Cyprus (Eastern Mediterranean)  **MED** | Loggerhead | 22 | 12-77 | 2011-16 | 36 | 52 | 4 | 13 | 4 | 6 |
|  | Green | 10 | 25-35 | 2016-17 | 30 | 0 | 0 | 4 | 2 | 0 |
| North Carolina, USA (Eastern Atlantic)  **ALT** | Loggerhead | 8 | 55-83 | 2016-17 | 0 | 0 | 0 | 1 | 4 | 0 |
|  | Kemp's Ridley | 10 | 23-41 | 2010-17 | 0 | 0 | 0 | 1 | 4 | 0 |
|  | Leatherback | 2 | 148-U | 2017 | 0 | 0 | 0 | 1 | 2 | 0 |
|  | Green | 7 | 6- 57 | 1993-2017 | 100 | 0 | 0 | 4 | 11 | 0 |
| Queensland, Australia (Coral Sea, Pacific)  **PAC** | Loggerhead | 3 | 5-71 | 2009-14 | 100 | 0 | 0 | 2 | 3 | 0 |
|  | Flatback | 4 | 10-23 | 2006-14 | 75 | 1 | 0 | 2 | 3 | 0 |
|  | Olive Ridley | 1 | 61 | 2016 | 0 | 0 | 0 | 0 | 1 | 0 |
|  | Hawksbill | 1 | 59 | 2016 | 0 | 0 | 0 | 0 | 2 | 0 |

**Table S1. Summary of marine turtles (n=102)** by sites, species, size (CCL: Curved Carapace Length cm; notch to notch), % macroplastic and synthetic particle ingestion presence. U=unmeasured due to damage. SCRFs = Synthetic Cellulose Regenerated Fibres

| **Origin** | **Group** | **FT-IR Identification** | **MED n= 121** | **ATL n= 19** | **PAC n= 29** |
| --- | --- | --- | --- | --- | --- |
|  | Elastomers | Chlorobutyl-536 Blair | 1 | - | - |
|  |  | Chlorobutyl-1051 Polycorp | 1 | - | - |
|  |  | Chlorobutyl-516 Blair | 5 | - | - |
|  |  | Ethyl-acrylate Vamac (Rubber) | 3 | - | - |
|  |  | Ethylene Propylene Diene Monomer (EPDM Rubber) | 16 | - | - |
|  |  | Hydronated Nitrile Butadiene Rubber (HNBR) | 19 | - | - |
|  |  | Nitrile-Butadiene Rubber (NBR) | 11 | - | 1 |
|  |  | Ethylene Propylene | 8 | - | - |
|  |  | Neoprene | 7 | - | - |
| **Synthetic** |  | Viton | 3 | - | - |
|  |  |  | 61.2% | 0% | 3.4% |
|  | Woven | Aramid Woven Fabric | 3 | - | - |
|  |  | Polyaramid, Kevlar® woven fibers | 3 | - | - |
|  |  |  | 4.9% | 0% | 0% |
|  | Plastics e.g. thermoplastics | Klockner Moeller 74 Relay Housing Piece2 | 1 | - | - |
|  |  | Nylon | - | 2 | - |
|  |  | Paraffin Wax and Polyvinyl Acetate Mixture | 2 | - | - |
|  |  | Polyacrylamide,Carboxy modified | 3 | 1 | 2 |
|  |  | Polyacrylic | - | 2 | 1 |
|  |  | Polyacrylate | 2 | - | - |
|  |  | Polycarbonate | - | 1 | - |
|  |  | Polyester Fibers | 4 | - | 3 |
|  |  | Polyethylene terephthalate | 6 | - | - |
|  |  | Polyethylene, chlorinated | 7 | - | 1 |
|  |  | Polypropylene | - | - | 1 |
|  |  | Plastised Polyvinyl Chloride (PVC) | - | 1 | - |
|  |  |  | 20.6% | 36.8% | 27.9% |
|  | Regenerated Cellulose | e.g. Rayon or Viscose | 7 | 12 | 20 |
|  |  |  | 5.8% | 63.2% | 68.9% |
|  | Rubbers | Natural Latex Rubber | 2 | - | - |
| **Non-synthetic** |  | Natural Rubber | 4 | - | - |
|  | Other | Zein | 3 | - | - |
|  |  |  | 7.4% | 0% | 0% |
| **Total:** |  |  | **121** | **19** | **29** |

**Table S2. Results from the subsample of isolated particles (n=169)** analysed using Fourier transform infrared spectroscopy (FT-IR) to determine their polymer make up from gut content residue samples of marine turtles


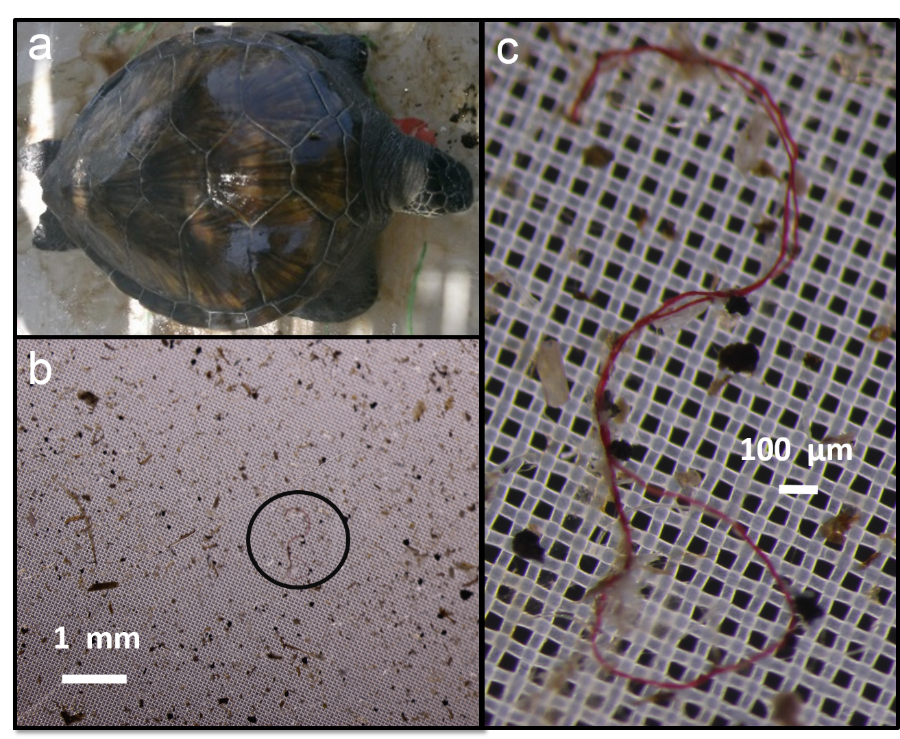


**Figure S1. Enzymatic digestion of marine turtle gut content** a) Stranded juvenile green turtle (CCL=33cm) from the North Cyprus coastline b) the gut content residue sample from the juvenile green turtle that has been enzymatically digested which has removed the majority of the biological material allowing the identification of suspected microplastics c) a microplastic fibre isolated from the gut content of the juvenile green turtle.


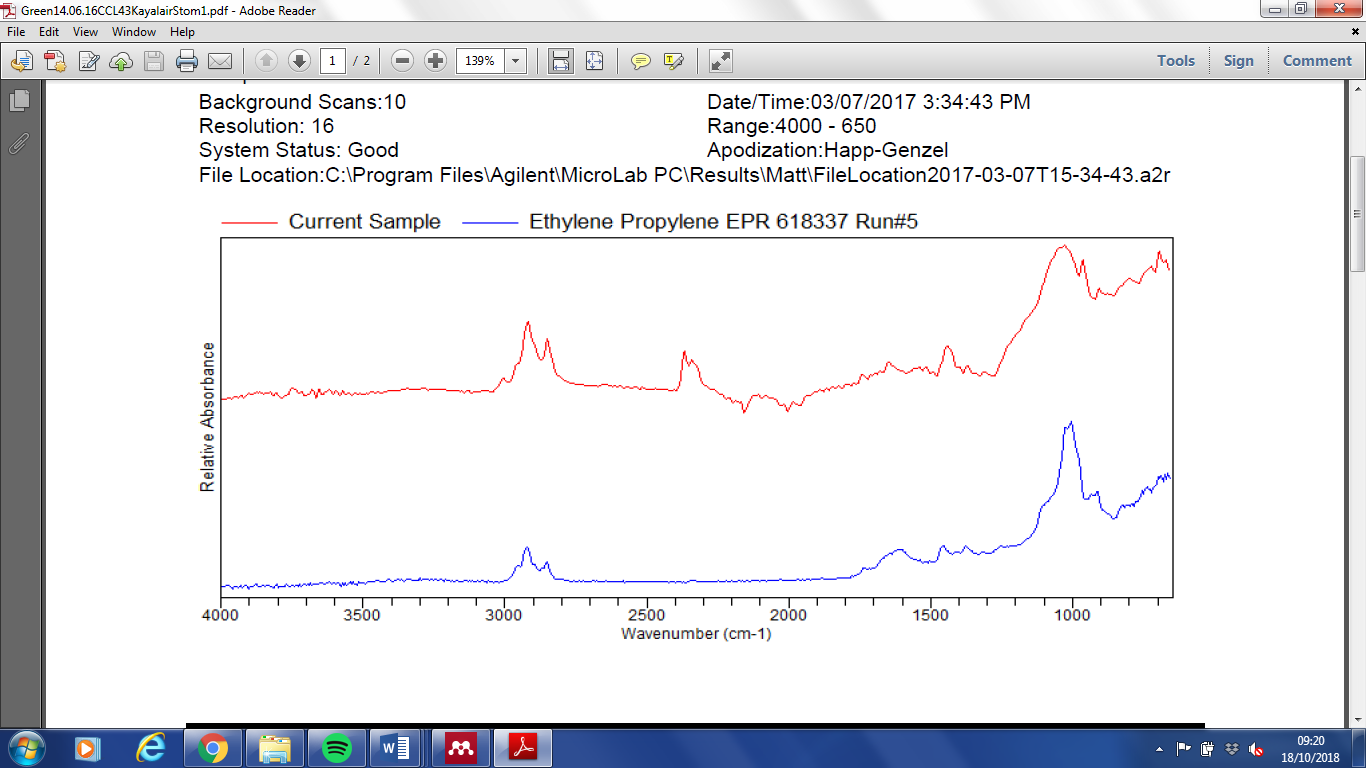


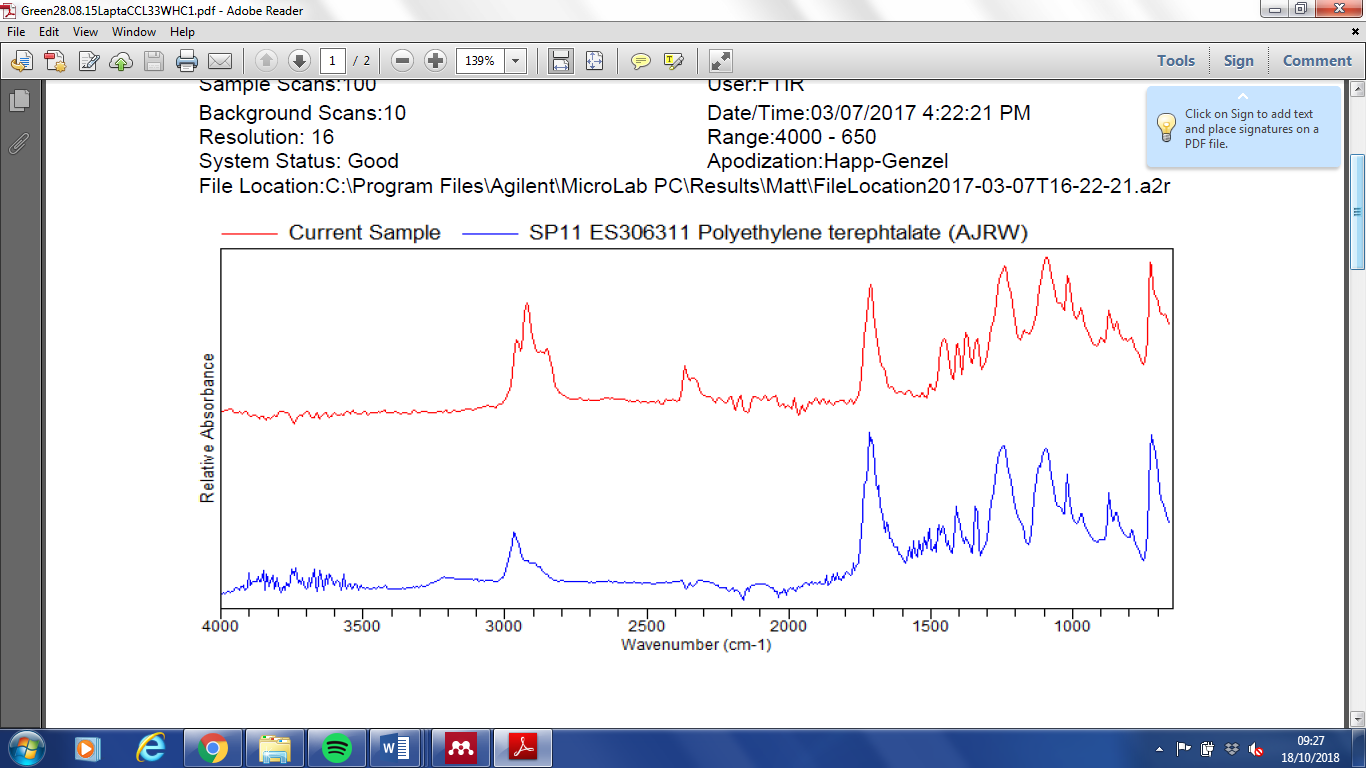


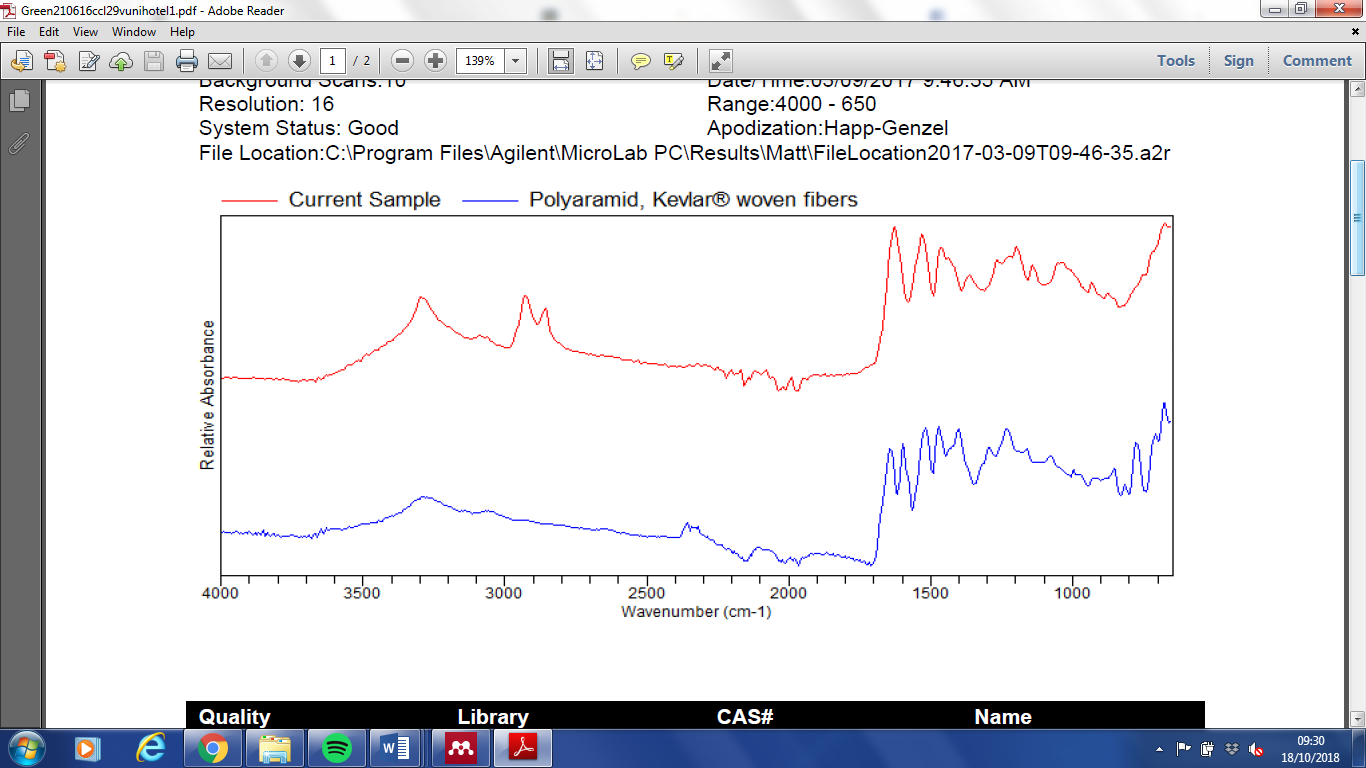


**Figure S2. Example spectra of synthetic particles from FTIR analysis** a) Ethylene propylene (91% confidence level) b) Polyethylene terephthalate (82% confidence level) c) Polyaramid (Kevlar woven fibres) (87% confidence level)

**Supplemental Methods**

**Necropsy and gut content analysis**

Turtles were subject to necropsy to determine the cause of death, and biometric parameters were taken (Wyneken, 2001). To determine marine litter ingestion we followed the Fulmar Protocol developed by van Franeker et al., (2011) for monitoring plastic ingestion in the seabird *F. glacialis* which has been recommended to be adapted to the Mediterranean loggerhead turtle by the Marine Strategy Framework Directive GES Technical Subgroup on Marine Litter (Matiddi et al., 2011). During necropsy the entire gastrointestinal tract was removed and initial contents was weighed and then rinsed through a 1mm mesh sieve. After this, the remaining matter in the sieve was emptied into trays for sorting. Dietary items were separated, weighed and identified, meanwhile suspected plastic or other marine debris was removed and stored for later analysis. A sample of 100ml of gut content residue and was collected from material that had passed through the 1mm mesh sieve. This approximated 5% of the supernatant liquid. This was later oven dried at 60˚c for 24 hours to enhance the efficacy of homogenizing the remaining biological material in later steps of the process.

**Enzymatic digestion**

The optimised enzymatic digestion protocol was developed for use on zooplankton material by Cole et al., (2014) and adapted for use on marine turtle gut content. Desiccated samples were lightly ground with a pestle and mortar, to increase surface area, and transferred into 50mL acid-washed, screw-top glass containers (to avoid contamination) with 15ml homogenizing solution (400mM Tris-HCI buffer, 60mM EDTA, 105mM NaCl, 1% SDS). Samples were homogenized physically by drawing and expelling the mixture through a 19G needle attached to a 10mL syringe, the insides of which were rinsed thoroughly with homogenizing solution to avoid the loss of any material. Samples were then incubated at 50˚C for 30 minutes before adding 375µl of 20mg/mL^-1^ of Proteinase-K. These were further incubated for 2.5 hours at 50˚C and 3ml 5 M sodium perchlorate (NaCLO_4_) was then added and samples shaken at room temperature for 30 minutes. Samples were homogenized a second time using a finer 21G needle, incubated at 60 ˚C for 30 minutes and then vacuum filtered on to pre-weighed 50µm mesh filters. Retained biological material was flushed copiously with Milli-Q water and the filters removed, covered and oven dried at 60 ˚C. To compensate for a greater amount of biological material having to undergo digestion from some gut content residue samples, filters were re-digested up to three times and each sample split between two to three 50 µm mesh-filters to prevent clogging and to more easily identify any microplastics present in these samples with higher amounts of biological material.

**Filter analysis**

Filters were analysed under a digital stereo microscope (Leica M165C). Microplastics particles were identified by assessing colour, uniformity of material and shape (Norén, 2007). These were then classified into three categories; fibres, fragments and bead. Microplastics were then further subcategorised into 11 colour categories (Black, Brown, Grey, White, Clear, Red, Orange, Yellow, Green, Blue, Purple). Particles were also measured; the length and width of fibres and the smallest diameter of fragments and beads, with examples photographed by a digital camera (Leica DFC295; Leica Suite Application Version 3.6.0).

**Size distribution of particles**

Fibre maximum length varied: MED: 1.40 ± 0.54mm (mean ± S.E); ATL: 2.87 ± 0.20; PAC: 2.85 ± 0.23mm. Fragment and bead diameter was smaller: MED: 0.07 ± 0.01mm (mean ± S.E); ATL: 0.31 ±0.04mm; PAC: 0.26 ±0.01 mm).

**Reducing contamination**

A number of measures were implemented throughout the procedure to limit the risk of contamination of the samples via air-borne particles or is present on equipment: sterile containers were used for sample collection, all apparatus used within the laboratory was acid-washed and/or rinsed thoroughly with Milli-Q before use (filtered to ensure to be particle free). Personal protective equipment (e.g. cotton lab coat/ nitrile gloves) was worn at all times and samples and all surfaces were wiped down with 70% ethanol prior to any work commencing. Work (e.g. vacuum pumping) was carried out inside a positive pressure laminar flow hood and equipment were covered wherever possible to minimize periods of exposure with the aim of preventing air-borne microplastics from settling on the samples. During enzymatic digestion all equipment was rinsed with Milli-Q and all pipettes and syringes were flushed with Milli-Q prior to use. Furthermore, procedural blanks, from which gut residue material was omitted, were run in parallel from the initial sampling at gut processing of the marine turtles and through the enzymatic digestion process. Three blank samples were performed alongside each digestion process of gut content material, for each round of sampling in each field site (ATL n=3; MED n= 6; PAC= 3) and treated in the same way as samples to help check for possible contamination. The analysis of these filters (n=12) showed minimal evidence of microplastic contamination with the presence of single fibres (n=9 cases) or very occasional fragments (n=3 cases) but no beads. These particles were noted to look qualitatively different to those on the gut content filters i.e. environmental contaminants presented in full vivid colour wheras the ones from gut content were visibly degraded with faded colours.

**Polymer Identification**

A sub sample (n=169) of these identified microplastics were analysed using Fourier Transform Infrared spectroscopy (FT-IR) (Agilent Cary 630 FTIR spectrometer; Agilent FTIR Spectral Library ePoly 8; PerkinElmer Spotlight 400 FT-IR Imaging System, MCT detector, KBr window; PerkinElmer Spectrum software version 10.5.4.738) to determine their polymer make up. When interpreting FTIR output the resulting spectra were compared to a spectral database from a number of polymer libraries to create match quality search scores, only those with scores 70% or greater and those considered to have reliable spectra matches (after visual inspection) were accepted (Figure S2.).

**References**

Cole M, Webb H, Lindeque PK, Fileman ES, Halsband C, Galloway TS (2014) Isolation of microplastics in biota-rich seawater samples and marine organisms. *Scientific reports*, **4**, 4528.

van Franeker JA, Blaize C, Danielsen J et al. (2011) Monitoring plastic ingestion by the northern fulmar Fulmarus glacialis in the North Sea. *Environmental Pollution*, **159**, 2609–2615.
